# Impact of High-Intensity Facial Electrical Stimulation Technology on Elevation of the Midface: A Prospective Study With Ultrasound

**DOI:** 10.1093/asjof/ojag103

**Published:** 2026-07-04

**Authors:** Steven Liew, Fadie Aziz, Nicky Lurie, Stefania Roberts

## Abstract

Nonsurgical skin tightening and lifting devices have gained significant popularity because of their ability to provide facial rejuvenation without the downtime and risks associated with surgery. Technologies utilizing high-intensity facial electrical stimulation (HIFES) in combination with synchronous radiofrequency (RF) have shown promise in achieving lifting effects in the upper and midface. The aim of this study was to evaluate the effectiveness of combined HIFES and synchronous RF treatment of the midface in producing lifting of the modiolus relative to 2 fixed anatomical landmarks: the superior tragal point (STP) and lateral canthal point (LCP). A prospective single-arm cohort study was conducted with 8 participants completing 4 weekly HIFES and synchronous RF treatments. The position of the modiolus was measured at 3 time points: pretreatment, immediately after the first treatment, and 3 months after the final session. The modiolus was localized with ultrasound, and distances to the STP and LCP were recorded. Means were compared using repeated-measure analysis of variance with pairwise *t*-tests; the Wilcoxon signed-rank test was performed as a robustness check. The modiolus demonstrated statically significant elevation in an oblique cephaloposterior vector toward the tragus. Modiolus–STP distance decreased from baseline to 3 months posttreatment (right: 0.56 cm, *P* < .01; left: 0.31 cm, *P* = .04). Modiolus–LCP distance also decreased (right: 0.28 cm, *P* = .06; left: 0.14 cm, *P* = .45), although not statistically significant. In this preliminary study, HIFES and synchronous RF treatment produced significant measurable lifting of the modiolus consistent with the anatomical pull of the zygomaticus complex. Larger studies with longer follow-up are warranted to further characterize these effects.

**Level of Evidence**: 4 (Therapeutic) 
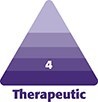

Facial aging is an inevitable process affecting all layers of the face, including skin, fat, connective tissue, muscle, and bone.^1-3^ Changes in the skin quality occur because of collagen and elastin degradation, whereas sarcopenia is responsible for muscle weakening resulting in sagging of overlying soft tissues with the effect of gravity.^4-6^ The muscles of the midface are interconnected by the midfacial superficial musculoaponeurotic system (SMAS), a connective tissue network supporting and serving as an attachment of midface muscles to the skin.^7^ Weakening of the midface muscles, specifically the zygomaticus, can result in midfacial descent with increased prominence of the nasolabial fold, jowl formation, and loss of jawline prominence.^8,9^

Facial aging can occur at different decades and progress at different rates between individuals of varying sex and ethnicity.^10,11^ The impact of one's appearance can be dissonant to how they feel or wish to portray themselves to the world and can have a significant impact on self-esteem.^12^ To date, a surgical facelift remains the gold standard in restoring and repositioning the facial elements described above with increasing acceptance, nuances, and safety.^13^

Despite this, potential patients still hold fear of the surgical invasiveness of facelift procedures and the associated risks and downtime. There has been an ever-increasing popularity of nonsurgical tightening and lifting procedures in the form of energy-based devices (EBDs) and facial threads.^13^ To meet the insatiable quest for a less invasive, efficacious and minimal-to-no-downtime rejuvenation procedure, the aesthetic market has seen an exponential expansion of EBDs with promises of effective tightening and lifting of sagging facial tissue. These EBDs are based on the generation of thermal energy from either synchronous radiofrequency (RF; monopolar or bipolar or both) or focal ultrasound, targeting the layers of the skin, superficial fat, and SMAS.^14^ The application of thermal energy aims to create soft tissue contraction, skin tightening, and improvement in skin quality. The literature supporting these devices is based on histological changes of soft tissue, including collagen and elastin staining and 2-dimensional (2D)/3-dimensional (3D) photography.^15^ No studies to date, as far as the authors are aware, involve any objective measurement of the lifting or skin tightening effect.

Most recently, a new concept of nonsurgically lifting the facial soft tissues has emerged in the form of the EMFACE device which aims to improve the resting tone of the elevators and mimetic muscles of the forehead and midface (EMFACE, BTL Aesthetics, Czech Republic). It works through utilizing a combined synchronous RF and high-intensity facial electrical stimulation (HIFES) to target the first 3 layers of the face, namely the skin, superficial fat, SMAS, and mimetic muscles.^16^ This synchronous energy is delivered through 3 electrodes applied to the facial muscles during weekly 20 min sessions in the office setting, over the course of 4 weeks. The HIFES targets the frontalis, zygomaticus major, zygomaticus minor, and risorius muscles.^17^ The muscles are induced with up to 250 energy impulses per second (250 Hz) in order to prevent any relaxation between the stimulus (supramaximal contraction).^18^ The synchronous RF works through heating the skin and fascial framework up to 40° to 42°, resulting in remodeling of collagen and elastin fibers and leading to increased elasticity and tightness.^19,20^ HIFES results in increased muscle tone and hypertrophy of the targeted mimetic muscles.^21,22^ The combination of these dual modalities creates a firmer overlying soft tissue envelope and an increase in the resting tone of the mimetic muscles of the upper and middle thirds of the face. This ultimately establishes lifting of the upper periorbita and the midface.^16,23,24^

The use of RF + HIFES has yielded positive results based anecdotally on patient satisfaction.^25-27^ Literature support for RF + HIFES has demonstrated increased density and number of myonuclei, brow-lifting effect, patient satisfaction, and wrinkle reduction.^28,29^ The senior author equally has seen the positive results above, based on clinical feedback from patients, photographic evidence and observation of the elevation of the midface and improvement in the position of the oral commissure (Figure 1). Driven by the absence of true objective measurements for the skin tightening and lifting effect of EBDs in the market, the authors decided to conduct a study to objectively quantify the clinical observation of the midface and oral commissure elevation in reference to a known anatomical landmark, the modiolus.

**Figure 1.**
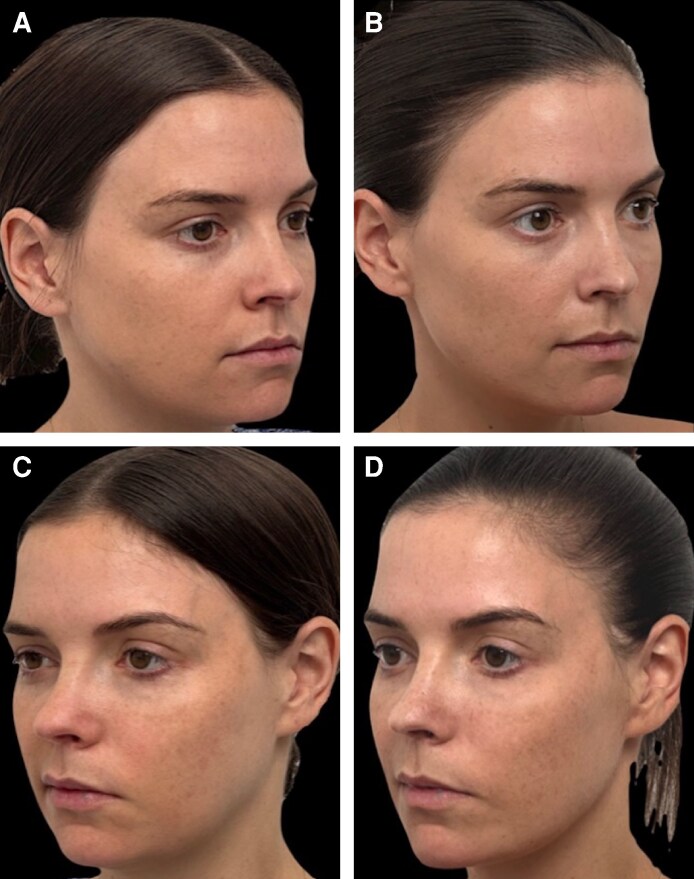
A 36-year-old female with left facial drop from Bell's palsy. (A, C) Images before treatment. (B, D) Appearance 15 weeks posttreatment with high-intensity facial electrical stimulation and radiofrequency. These images showcase a more lifted upper periorbita, lifted midface with improved definition and projection, uplifted oral commissures bilaterally, and improved jawline.

The modiolus is an important anatomical landmark at the angle of the mouth.^30-32^ It is an important fibromuscular structure next to the oral commissure, inserted by a confluence of mimetic muscles including the lip elevators (zygomaticus major and risorius), buccinator, orbicularis oris, the lip depressors (depressor anguli oris), and pars modiolus of the platysma muscle.^33,34^ The main targeted mimetic muscles of the midface with RF + HIFES are the zygomaticus major, minor, and risorius.

We hypothesize that RF + HIFES treatment enhances the tone of the lip elevators, creating a more favorable upward muscular balance at the modiolus relative to the lip depressors. This theoretically should create a change in the position of the modiolus as visualized by ultrasound and measured in relation to the 2 fixed anatomical facial landmarks, the lateral canthal point (LCP) and superior tragal point (STP; Figure 2).

**Figure 2.**
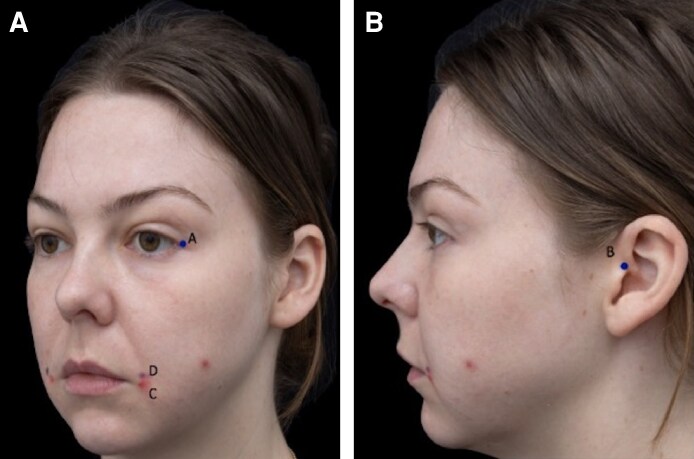
A 30-year-old female demonstrating anatomical landmarks used in this study. (A) Lateral canthal point. (B) Superior tragal point. (C) Modiolus position before treatment. (D) Modiolus position after treatment.

## METHODS

This study was a prospective single-arm cohort study conducted in a private clinic setting. Ethics approval was obtained through the local ethics committee.

The St Vincent's Hospital Human Research Ethics Committee, (Sydney, Australia, ethics approval number ETH00825) and was performed according to the Declaration of Helsinki.^35^

### Study Sample

Participants were recruited on a voluntary basis and were considered suitable for inclusion if they satisfied the inclusion criteria. This included nonpregnant patients aged 35 to 60 who were willing to provide written consent for participation in the study, including the use of their images and personal data for research purposes. Exclusion criteria included patients who had any facial surgery 12 months before the study or had any facial trauma or scarring from previous facial surgery or trauma that may distort or affect facial muscular anatomy. Additional exclusion criteria included patients with metallic implants in the face, cardiac defibrillators, and those patients who had any nonsurgical facial treatment (including all types of facial injectable and EBDs) 3 months before the study or who planned to receive any other form of nonsurgical or surgical facial treatment before completion of final measurements.

### Treatment, Ultrasound Mapping, and Measurement Conducted

Participants underwent a total of 4 treatment cycles with RF + HIFES as per the treatment protocol with treatment cycles occurring once weekly. Each treatment session lasted 20 min. Measurements for this study were made at 3 time points—before commencement of treatment, immediately following first treatment, and at 3 months following the final treatment.

**Figure 3.**
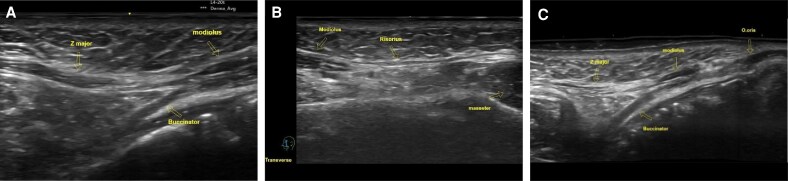
(A) Ultrasound image with the probe in transverse, the zygomaticus major and the buccinator muscles are shown as they insert into the modiolus. (B) Ultrasound image with the probe held in transverse showing a thin hypoechoic band. The risorius muscle arises from the anterior border of the masseter muscle with insertion into the modiolus. (C) A panoramic view with the probe in transverse showcases the ultrasonic location of the modiolus based on the convergence of zygomaticus major, buccinator, and the orbicularis oris muscle.

Localization and marking of the modiolus was performed by an investigator with experience in ultrasound. All measurements were performed with the patient positioned supine at 45°. The modiolus was visualized with Venue Go ultrasound machine (GE Healthcare) using the Linear probe L4-20t-RS (3-20 MHz). The modiolus was identified by locating the insertion of zygomaticus major, risorius, buccinator, and orbicularis oris, noting these muscles exist in different anatomical planes (Figure 3). In each patient, the latter 4 muscles were identified on ultrasound and traced to their confluence on the modiolus. Zygomaticus major was located with the probe transversely on the zygomatic arch. The probe was then rotated in a clockwise direction with the toe of the probe in the direction of the oral commissure. Zygomaticus minor was visualized in an oblique plane. Risorius, when present, was identified with the midpoint of the probe on the anterior border of the masseter. Using a systematic sweep from the mandibular body inferiorly to the maxilla superiorly, a thin, hypoechoic band between the anterior border of the masseter and the posterior fibers of the depressor anguli oris representing the risorius was sought.^36^ Buccinator was identified with the probe in transversely along the inferior border of the mandible and traced anteriorly to the modiolus. Orbicularis oris was identified with the probe in transversely on the upper lip. Where the 4 described muscles merged led to the location of the modiolus (Figure 3). The modiolus was further confirmed by placing the midpoint of the probe in transverse over the oral commissure and was seen as a compact hypoechoic area both in the transverse and longitudinal plane.^37^ The position of the facial artery in relation to the modiolus was also identified in each of the subjects to replicate the marking of the modiolus at each stage of the study.

Utilizing the above methodology, the midpoint of the modiolus was marked with a marker on each side of the face before the first RF + HIFES treatment. This formed the baseline modiolus location. Immediately following the first treatment, and at 3 months following the fourth treatment, the new modiolus positions were marked with a different color marker.

Measurement of the shift in position of the modiolus following the above treatments was measured by 2 of the investigators. The position of the modiolus was measured from 2 fixed anatomical landmarks—the LCP and the STP. The LCP is defined as the junction at which the upper and lower eyelids meet laterally. The STP is defined as the point at which the skin of the tragus meets the root of the helical rim (Figure 2). The modiolus-to-LCP and modiolus-to-STP distances were measured with a standard plastic disposable ruler that allowed it to be placed on the surface and gently molded to follow the curvature of the face. At each landmark, the center of the markings of STP, LCP, and modiolus is used. Each distance was measured twice by each of the 2 investigators, and given the results correlated well between investigators, a mean was used for analysis.

### Imaging

Standardized 2D and 3D imaging was performed with all participants for the 3 time points in the study. 3D imaging was performed with Quantificare LifeViz Mini (France).

### Analytic Process

Data were analyzed using IBM SPSS Statistics for Mac, Version 29.0 (IBM Corp., Armonk, NY). An intraclass correlation coefficient (ICC) was calculated to assess interinvestigator reliability between the 2 independent assessors; where good agreement was demonstrated (ICC ≥ 0.75), the mean of the 2 investigators’ measurements was used for analysis. Given data were normally distributed and maintained the sphericity assumption, a repeated-measure analysis of variance (ANOVA) was utilized with post hoc pairwise *t*-tests (with Bonferroni correction) to compare means. Our post hoc analysis was performed for each side of the face to compare the means for each distance (modiolus-to-LCP and modiolus-to-STP) over the 3 time points (pretreatment, post-first treatment, and postfinal treatment). A 2-tailed *α* level of .05 was considered statistically significant.

We performed 2-sided tests to test the hypothesis that treatment with RF + HIFES results in a change in the position of the modiolus with respect to the LCP and STP. Given the small sample size, we also performed a Wilcoxon signed-rank test as a robustness check for our results.

## RESULTS

A total of 8 patients were enrolled in the study, with 1 patient being lost to follow-up (did not present for final measurement at 3 months). The study recruited 8 healthy participants, all females from 39 to 57 years old with a mean age of 42. Results for each participant are included in Table 1. ICC between the 2 investigators performing measurements was 0.99 (95% CI, 0.98-0.99, *P* < .01), indicating excellent correlation (>0.9). Therefore, a mean of measurements from the 2 observers was used for analysis. Data were normally distributed as confirmed with the Shapiro–Wilk test, whereas the Mauchly test confirmed that the sphericity assumption of the data was true. We appreciate these tests are less sensitive for small sample sizes, and as such, we utilized a Wilcoxon signed-rank test as a robustness check.

**Table 1.** Modiolus Landmark Distances by Patient, Side, and Time Point

Participant	Side	Modiolus–LCP distance (cm)			Modiolus–STP distance (cm)		
		Pretreatment	Immediate post-first treatment	3 months postfinal treatment	Pretreatment	Immediate post-first treatment	3 months postfinal treatment
1	Right	7.03	6.70	6.93	11.48	10.65	10.93
	Left	7.13	6.88	6.73	11.45	11.23	10.60
2	Right	6.90	6.23	6.48	10.35	9.85	9.80
	Left	7.30	6.73	7.00	10.33	10.05	9.50
3	Right	7.18	6.63	6.95	10.30	10.05	10.03
	Left	7.43	6.98	7.05	10.73	10.43	10.15
4	Right	7.30	6.95	7.28	10.48	10.35	10.18
	Left	7.68	7.38	7.50	10.98	10.55	10.23
5	Right	7.33	6.78	6.90	10.30	10.03	9.90
	Left	7.33	7.35	6.93	10.33	10.45	9.88
6	Right	7.93	7.43	8.10	10.60	10.58	10.30
	Left	7.90	7.55	8.10	10.23	10.15	10.13
7	Right	7.00	6.60	7.03	10.93	10.85	11.08
	Left	7.33	6.88	6.80	10.95	10.58	10.53
Mean (SD)	Right	7.24 (0.34)	6.76 (0.37)	7.10 (0.50)	10.63 (0.43)	10.34 (0.37)	10.32 (0.50)
	Left	7.44 (0.26)	7.11 (0.10)	7.16 (0.48)	10.71 (0.45)	10.49 (0.38)	10.15 (0.38)

LCP, lateral canthal point; SD, standard deviation; STP, superior tragal point.

**Figure 4.**
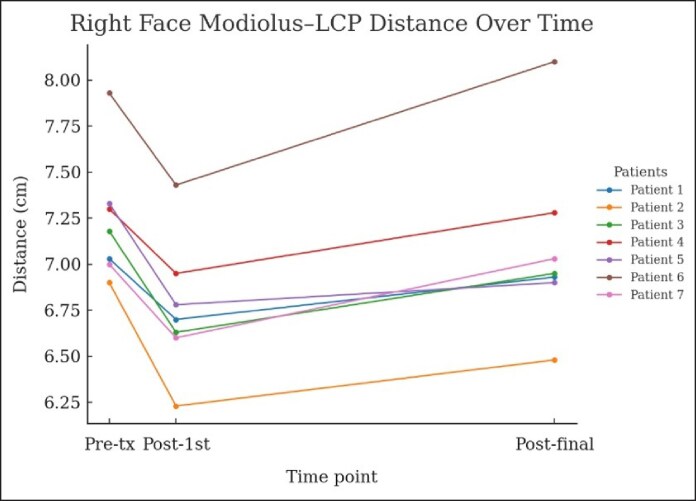
Modiolus position for the right face with respect to the lateral canthal point (LCP).

**Figure 5.**
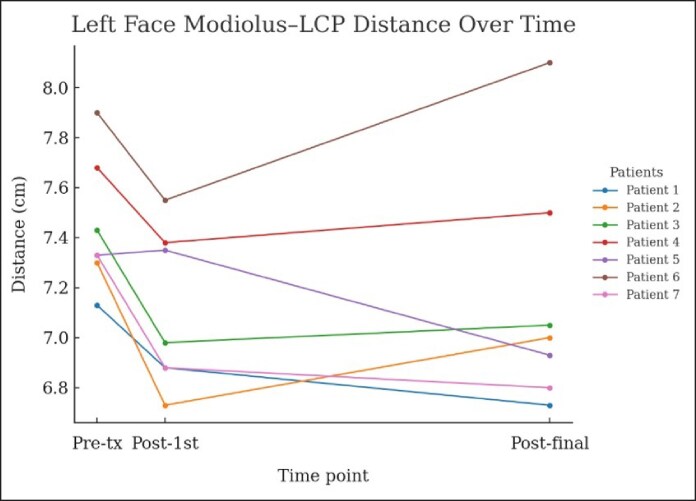
Modiolus position for the left face with respect to the lateral canthal point (LCP).

**Figure 6.**
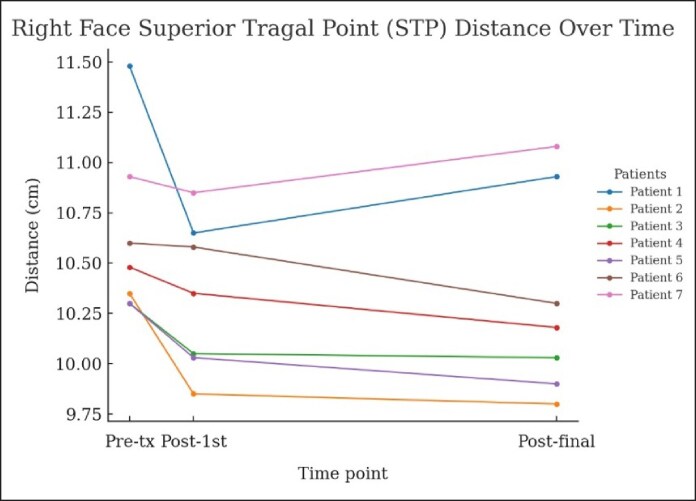
Modiolus position for the right face with respect to the superior tragal point (STP).

**Figure 7.**
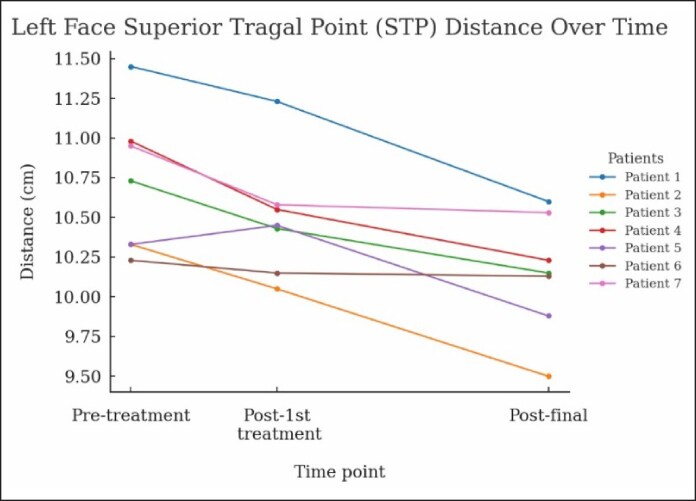
Modiolus position for the left face with respect to the superior tragal point (STP).

Using repeated-measure ANOVA, partial eta-squared ($\eta_{\rho }^{2}$) = 0.99 with *P* < .01, indicating true variance among results across the 3 time points for STP and LCP. Results from post hoc analysis using pairwise *t*-tests are included in Table 2. Using the Wilcoxon signed-rank test as a robustness check, *P*-values at level *P* < .05 corresponded with those for the pairwise *t*-test indicating equivalent results if the data is treated nonparametrically. Therefore, we included *P*-values from the pairwise *t*-tests in Table 2. The measurements of modiolus position for each patient comparing pretreatment to postfinal treatment are presented in Figures 4-7.

**Table 2.** Comparison of Mean Modiolus Landmark Distances Across Treatment Time Points

Measurement (mean)	Side	Pretreatment	Immediate post-first treatment	3 Months postfinal treatment	Mean difference (95% CI) with *P*-value		
					Pre- vs post-first treatment	Pre- vs 3 months postfinal treatment	Post-first treatment vs 3 months postfinal treatment
Modiolus-LCP distance (cm)	Right	7.24	6.76	7.10	0.48* (0.33-0.63) *P* < .01 0.33* (0.10-0.57) *P* = .01	0.14 (−1.4 to 0.43) *P* = .45 0.28 (−0.12 to 0.58) *P* = .06	−0.34 (−0.55 to −0.12) *P* = .01 −0.05 (−0.44 to 0.34) *P* = 1
	Left	7.44	7.11	7.16			
Modiolus-tragus distance (cm)	Right	10.63	10.34	10.32	0.29 (−0.05 to 0.65) *P* = .1 0.22 (−0.011 to 0.46) *P* = .06	0.31* (0.02-0.61) *P* = .04 0.56* (0.23-0.90) *P* < .01	0.02 (−0.24 to 0.28) *P* = 1 0.34* (0.04-0.66) *P* = .03
	Left	10.71	10.49	10.15			

LCP, lateral canthal point.

^*^The mean difference is significant at the *P* = .05 level.

For the left face, the mean modiolus-to-LCP distance was 7.44 cm (standard deviation [SD] 0.26) pretreatment compared with 7.16 cm (SD 0.48) post-fourth treatment with a difference of 0.28 cm (95% CI, −0.12 to 0.58, *P* = .06) indicating a nonsignificant change. The mean modiolus-to-LCP distance post-first treatment was 7.11 cm (SD 0.10) with a difference of 0.33 cm (95% CI, 0.10-0.57, *P* = .01) compared with pretreatment, indicating a significant change. The mean modiolus-to-STP distance was 10.71 cm (SD 0.45) pretreatment compared with 10.15 cm (SD 0.38) post-fourth treatment with a difference of 0.56 cm (95% CI, 0.23-0.90, *P* = .004) indicating a significant change. The mean modiolus-to-STP distance post-first treatment was 10.49 cm (SD 0.38) with a difference of 0.22 cm (95% CI, −0.11 to 0.46, *P* = .06) compared with pretreatment, indicating a nonsignificant change.

For the right face, the mean modiolus-to-LCP distance was 7.24 cm (SD 0.34) pretreatment compared with 7.10 cm (SD 0.50) post-fourth treatment with a difference of 0.14 cm (95% CI, −1.4 to 0.43, *P* = .45) indicating a nonsignificant change. The mean modiolus-to-LCP distance post-first treatment was 6.76 cm (SD 0.37) with a difference of 0.48 cm (95% CI, 0.33-0.63, *P* < .001) compared with pretreatment, indicating a significant change. The mean modiolus–STP distance was 10.63 cm (SD 0.43) pretreatment compared with 10.32 (SD 0.50) post-fourth treatment with a difference of 0.31 cm (95% CI, 0.02-0.61, *P* = .04) indicating a significant change. The mean modiolus–STP distance post-first treatment was 10.34 cm (SD 0.37) with a difference of 0.29 (95% CI, −0.05 to 0.65, *P* = .1) compared with pretreatment, indicating a nonsignificant change.

## DISCUSSION

At 3 months post 4 cycles of RF + HIFES, there was a general trend of elevation in the position of the modiolus with respect to both the lateral canthus and tragus (Figures 4-7). This is represented by a general trend of reduction or shortening of distances between modiolus to LCP and STP which is more pronounced for the STP. The results from Table 2 show a mean elevation of 0.31 cm on the left (*P* = .04) and 0.56 cm on the right (*P* < .01) for the STP, when comparing pretreatment to postfinal-treatment positions at 3-month follow-up. Both findings reached statistical significance (*P* < .05). With respect to the LCP, modiolus elevation had a mean of 0.14 cm on the left (*P* = .45) and 0.28 cm on the right (*P* = .06). Neither measurement reached statistical significance (*P* ≥ .05).

This observation of more pronounced and statistically significant lifting toward the tragus reflects the direction of pull of the targeted muscles with RF + HIFES. The 3 targeted muscles (zygomaticus minor and major and risorius), arise and insert into the modiolus in a more oblique vector. With stimulation of these muscles and increase in muscular tone posttreatment, the final vector of pull of the modiolus will be in a more oblique direction as demonstrated in the findings (Table 2). This is in contrast with the LCP to modiolus vector, which is more vertically orientated than that of the direction of pull of the targeted muscles. This offers an explanation for the lack of statistically significant findings when assessing elevation with respect to the LCP.

At 3 months postfinal treatment, the modiolus was found to be lifted even further toward the tragus (STP–to-modiolus distance) compared with the measurement immediately after the first treatment, albeit not statistically significant (Table 2). This may be explained by the cumulative effect of the RF, resulting in tightening of the soft tissue and fibrous network surrounding the modiolus, and the increase in strength and thickness of the targeted lip elevators.^22,38-40^ This is the same fibrous network extension that runs from the mimetic muscles to the overlying SMAS, superficial fat pad, and dermis.^41^ This tightening, together with the increased structural muscular thickness and muscle tone, creates further lifting of the modiolus.^42-44^ Interestingly, this was not seen with the LCP measurement which showed attenuation of the lifting effect when comparing measurements immediately post-first treatment to measurements 3 months postfinal treatment. We believe that immediately after treatment, there is a rise in muscle tone of not only the directly targeted muscles (zygomaticus minor, major, and risorius) but also the more vertically orientated lip elevators. This would result in a transient reduction of LCP measurement seen early but not sustained. This is best seen in the measurement for Patient 6, an outlier with an increase in LCP-to-modiolus distance on both sides of the face at 3 months. At 3 months, the structurally thicker and presumably stronger muscular tone of the 3 targeted muscles shifts the orientation of the modiolus more laterally and obliquely, thus resulting in the increase in the overall distance measured from LCP to modiolus.

Patient 7 is also an anomaly with the right side of the STP-to-modiolus distance showing an immediate reduction followed by an increase at 3 months. This is the only patient that experiences such deviation from the general trend of modiolus elevation toward the tragus. We believe this may be because of ultrasound measurement variability—including probe angulation, tissue compression, and challenges in consistent modiolus localization—rather than a true treatment-related response.

**Figure 8.**
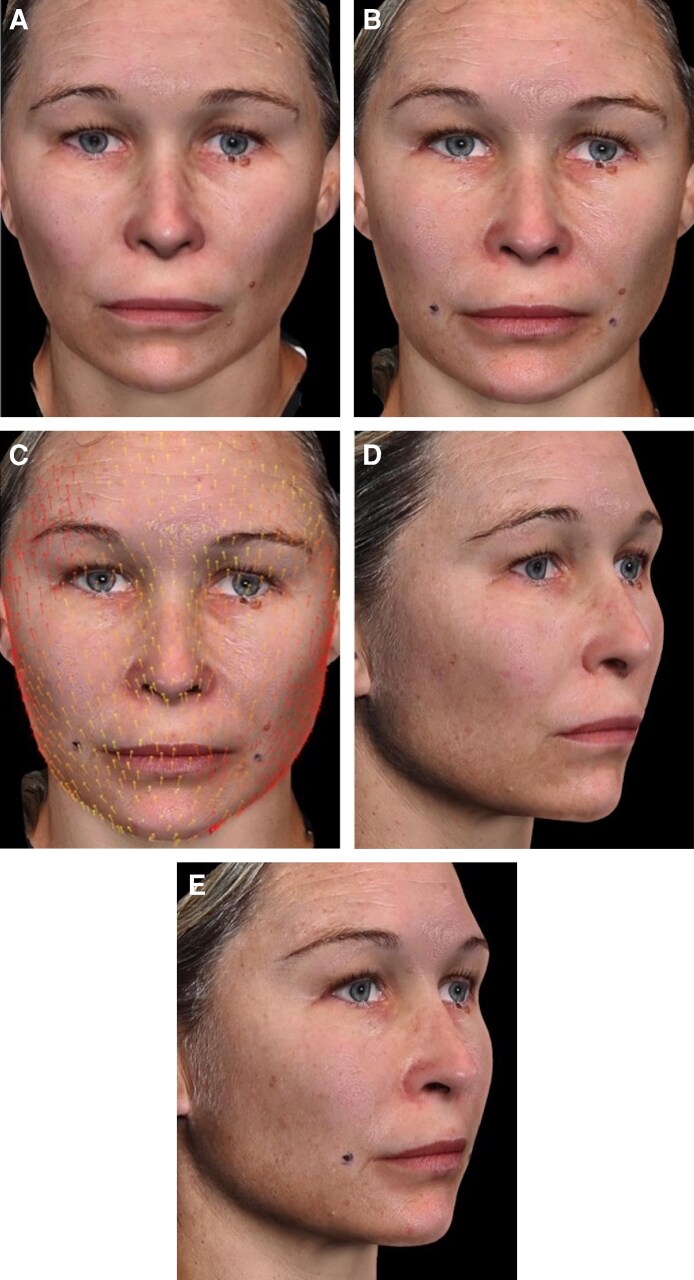
Three-dimensional image with Quantificare LifeViz Mini (France). (A) A 45-year-old female patient, before treatment. (B) Three months following 4 treatments of radiofrequency + high-intensity facial electrical stimulation, showcasing bilateral elevated eyebrows and fuller midface, with elevation and slight widening of the oral commissures. (C) Three months posttreatment, showcasing directional changes of the skin topography (yellow and orange arrows) of the midface and oral commissure in the cephaloposterior vector. (D, E) Three-dimensional images before treatment compared with 3 months posttreatment.

The study provides objective evidence that combination of intense and sustained electrical stimulation of the elevators of the lip together with bulk RF heating of the soft tissues can physically lift the modiolus by up to 0.5 cm. This explains the clinical improvements seen by most patients following RF + HIFES treatment (Figures 1, 8).

The improvements are, namely, a fuller and more elevated midface appearance, elevated upper periorbital tissue, and a lifted oral commissure.^16,45,46^ The modiolus forms the attachment of the orbicularis oris muscle and is just adjacent to the oral commissure. With its new lifted position following RF + HIFES, together with tighter soft tissue, the oral commissure can potentially be lifted accordingly. With an increased resting tone of the targeted lip elevators, especially the zygomatic complex muscles, the overlying interconnected fibrous network that connects the malar fat pad to the dermis will naturally be lifted, creating a slightly fuller midface appearance.^47^

We recognize that there are a few limitations of this study. First and foremost is the small sample size. Given no previous similar study, no power calculation was made because of the absence of a known effect size. Future studies with larger sample sizes would be more sensitive in detecting differences and provide more robust evidence, especially with the use of multivariable modeling. In this study, we used a protocol of 4 treatments as per the company recommendation, and this forms current clinical practice. It would be interesting to compare alternate treatment protocols (eg, 4 treatments vs 6 vs 12) to see which can provide the most effective lifting. In addition, an extension of study follow-up beyond the 3 months could establish the duration of the lifting effect, allowing a more objective recommendation to the patients for retreatment frequency.

In summary, this study represents the first anatomical and objective evidence showcasing the effectiveness of HIFES and synchronous RF technology in producing midface lifting through measurable changes in the position of modiolus as visualized by ultrasound.

## CONCLUSIONS

Targeting and strengthening the facial elevators opens a new paradigm of thinking for skin-lifting devices. Rather than simply treating the end effects of facial aging, this new technology aims to target one of the underlying causes of aging process, namely reduction in the tone and strength of the elevator muscles.

This study represents the first objective anatomical evidence supporting midfacial lifting with an EBD based on a new technology targeting tissue beyond the skin.

Further studies with larger sample sizes, longer follow-up, and exploration of different treatment protocols would be valuable in better examining the durability and clinical significance of these changes.
